# Ten simple rules for scientists engaging in science communication

**DOI:** 10.1371/journal.pcbi.1011251

**Published:** 2023-07-20

**Authors:** Brittney G. Borowiec

**Affiliations:** 1 Department of Biology, University of Waterloo, Waterloo, Canada; 2 Freelance science writer and editor (website: www.bgborowiec.com); Carnegie Mellon University, UNITED STATES

This is a *PLOS Computational Biology* Methods paper.

There are clear moral and professional imperatives for scientists to participate in communicating their research to the public [1–3]. Many scientists want to share their findings and excitement of science or encourage interest and appreciation of their work. They may want to improve the public’s understanding of their field or dispel misinformation. They may also be curious about how their work is viewed by society and benefit from interacting with new ideas and perspectives. Increasingly, science communication is a formal responsibility for researchers as funding bodies and institutions encourage or even require scientists to engage in science communication or outreach work (Broader Impacts for National Science Foundation grants, NSERC’s PromoScience program, etc.).

Despite there being many excellent reasons for scientists to engage in science communication, they often lack the tools to do so. Explicit training in nonexpert communication remains uncommon in graduate programs (though this is improving; [4]), as programs typically prioritize the development of scientist-to-scientist communication skills such as conference presentations and peer-reviewed articles. Scientists frequently cite lack of training and/or confidence in their science communication skills as a barrier to their participation in public-facing activities [4–8]. At the same time, there are ample opportunities for scientists to engage in science communication as a part of their official duties, as a secondary career (freelancing), or as a volunteer. As experts in their field, scientists can contribute through interviews in the media, writing op-eds or books for the popular press (see [9] for specific guidance on writing popular science books), public seminars or interactive events, social media posts (see [10] for specific suggestions on how to get started on Twitter as scientist), and other modalities.

I am a postdoctoral fellow and freelance science writer and editor. Here, I describe 10 simple rules for planning, developing, and evaluating science communication activities. Though I focus on scientists communicating with nonscientists, much of the advice applies to other forms of science communication such as expert-to-expert communication (e.g., talks and posters at conferences). As my goal is to guide inexperienced scientist-science communicators through the practical basics of getting started with science communication, the rules are ordered to encourage a step-by-step process (but note the integrative nature of science communication activities, Rule 10).

## Rule 1: Define your goals

Science communication activities can serve a variety of functions, including informing others about a topic, discussing a potential solution to a problem, advocating for a policy change, or correcting misinformation. The first step of any science communication activity is establishing the ultimate goal(s) of the activity, as all subsequent decisions to be made (selecting a mode of engagement, crafting the headline, calling for a specific action, etc.) will centre these goals. As science communication is essentially a service to an audience [11], an effective approach for defining activity goals is to consider how the activity will impact its potential audience and wider society in which that audience lives. Note that goals are key to post-activity evaluation and improvement, and so should be specific and testable or quantifiable whenever possible (via a post-activity survey of audience knowledge, engagement metrics such as attendance, views, retweets, etc.).

## Rule 2: Figure out who and where your audience is

“Know your audience” is a fundamental concept for science communication because the intended audience informs many decisions around activity design, completion, and evaluation. For example, activities that effectively engage young students (e.g., a “Do-It-Yourself” science experiment with household items) are unlikely to engage professional adults in the same way due to different interests and concerns. Science communication activities should be targeted towards an audience that is as discrete and specific as possible (e.g., compare 8-year-old children who have seen a specific movie versus all children studying at a specific elementary school). It is much easier to craft a clear message and leverage common ground between the communicator and the audience, both of which can increase audience engagement and therefore activity success, with a smaller group of people compared to the nebulous concept of “the general public.”

Determining and characterizing which public(s) will be served by an activity is essential for good science communication practice. For scientists partnering with established organizations such a media outlet (e.g., writing an op-ed) or institution (e.g., consulting on a new museum exhibit, visiting a classroom), the audience will already be well-defined and understood by the organization. For scientists leading their own science communication activities, some investigative work is often necessary to discern the beliefs, attitudes, concerns, values, and needs of the target audience. This can include thought exercises (e.g., an audience profiling exercise where they identify potential audience members, their interests, concerns, relationships, and other characteristics) [12], review of previous activities that appear to target the same audience, or, ideally, direct interaction with members of the potential audience. For inexperienced scientist-science communicators, this work is valuable for developing the ability to “see the world” through the eyes of their audience (empathy), a key skill for effective science communication [13].

Once the target audience is determined, all aspects of the activity can be customized to fit that specific audience. Different audiences may find the same topic exciting, boring, controversial, or even in bad taste. They may also have different concerns or questions about a particular topic, react differently to the same message, have different understandings of key terms, or be predisposed to respond with certain actions (e.g., see the Global Warming’s Six Americas project for an example of how different segments of the general public view and respond to climate change) [14].

In addition to understanding the “who” of the audience, it is also important to consider how to best engage with that audience (“where”). Choosing where a science communication activity occurs involves consideration of many factors and, at times, competing interests. First and foremost, an activity must be as accessible as possible to its target audience. For example, a “pub night” activity (i.e., a scientist presents to and/or interacts with patrons at a local restaurant that serves alcoholic beverages) beginning at 10 PM will exclude most school-aged children and possibly their parents. It may also exclude adults who avoid crowded indoor spaces, alcohol, or any other features of the pub. Conversely, a pub night may be a popular event for students attending the local college and residents of the neighbourhood who frequently visit the business. The venue may also be attractive for other reasons such as having an interesting atmosphere or being more cost-effective than a local conference centre.

Finding a place to do science communication can be difficult, especially for less experienced science communications with small networks and few contacts. Some places where scientists may look to get started can include offices at their institution (many have outreach offices or personnel), organizations in their community (especially if that organization has a relationship with the scientist’s institution), media outlets or their editors (either as a contributor or potential future source), or social media (which has little to no barrier to entry).

## Rule 3: Pick an arena that suits your goals and plays to your strengths

The communicator should select a mode of engagement that serves the activity’s goal(s). Some modes of engagement, such as a newspaper op-ed describing new research findings, offer a largely one-way mode of communication and are suited for goals such as “informing.” Other modes of engagement, such as town halls and some forms of social media, allow for two-way communication via prolonged interaction and/or discussion between communicator and audience and are better positioned to achieve goals related to “advocating” or “discussing.” Different communication modalities are likely to have different results. For example, two-way forms of communication such as dialogue that enable deeper audience participation in the activity are more likely to result in real and prolonged behavioural changes in the audience [15].

When possible, science communicators should also choose a mode of engagement that they feel confident participating in and that benefits from their personal strengths. For example, infographics greatly benefit from communicators with graphic design or photography skills, and communicators with excellent conversation skills may enjoy radio or podcast interviews.

It should be noted that another aspect of choosing an arena is deciding which battles are worth fighting in the first place. Science, and therefore science communication, always has been and always will be political. Scientists, especially those affiliated with specific institutions, need to be careful in how they navigate institutional policies (which may include limits on how and to whom they speak—e.g., the “muzzling” of scientists under a previous Canadian government [16]), especially if they plan on discussing even vaguely political and/or controversial topics.

## Rule 4: Come up with a clear headline message

There are several excellent templates for crafting an effective science communication message (The COMPASS Message Box, Olson’s And/But/Therefore, the Union of Concerned Scientists’ basic science/new finding/implications approach, etc.) [3,17–19]. These templates function to aid communicators in differentiating their topic from their message, which can be particularly challenging for scientists as they are accustomed to discussing nuanced concepts with their scientific peers. These templates also aid communicators in centring the perspectives of their audience and in framing the activity in a way that resonates as engaging and valuable to that audience.

Regardless of whether the activity is a 1-minute video, a popular science book, a multiday event, or something else, it is key to develop a headline. Ideally, this is a simple, clear, and short phrase (or phrases) that describe the most important piece(s) of information for your audience to absorb from the activity. Put another way, if the audience remembers only one idea, concept, or opinion from the activity, what should it be?

Having clear headline is essential for short-format science communication activities (e.g., Tweets), as it may be the only information and context that will be available to the audience. But a good headline is also beneficial for content-heavy activities where bits and pieces of information will be remembered by different members of the audience. The strategic advantage of a headline is that it ensures that a very specific piece of information is absorbed by as many audience members as possible. This approach helps structure the activity (the headline should be reinforced at several points during the activity), can inhibit misinterpretation or the spread of misinformation (a short headline is easier to understand than a graph or dense abstract, for example), and facilitates broader sharing of the activity’s core message by making it more shareable by participants (similar to how headlines function in journalism or on social media). For inexperienced science communicators, crafting a headline is also valuable for skill development, as it challenges the communicator to think deeply about their goals and adapt them as appropriate.

## Rule 5: Beware of jargon

Jargon is specialized language used between two or more experts as shorthand for complex ideas [3,20]. When used between an expert and nonexpert, jargon loses its value and can function as a barrier to effective communication [20–22]. Science communication activities cluttered with technical jargon and acronyms (“alphabet soup”) are ineffective and are likely to be ignored by their prospective audiences.

Scientists struggle in both identifying jargon terms [23,24] and in balancing being easily understood with retaining a personally acceptable level of precision, conciseness, and authority. A keen awareness of their audience is a communicator’s best tool for identifying what is and is not jargon. It also is essential knowledge for developing audience-specific strategies for handling jargon such as replacing it with more accessible terms, metaphors, or analogies (e.g., DNA’s “double helix” becomes a “ladder” or “spiral staircase”) or providing audience-appropriate definitions and context. It should be noted that the goal should not be to purge all jargon from all science communication. Jargon is not always bad. In fact, thoughtful inclusion of jargon may contribute towards goals such as informing or educating the audience in a topic or building relationships with the audience by initiating them into the “club” of expert jargon users.

## Rule 6: Show your audience why they should care

As a science communicator, you want your audience to value the headline message enough to remember it and, ideally, repeat it to others. It is key to remember that knowledge for knowledge’s sake does not motivate all audiences in the same way and that curiosity-driven inquiry, especially in a professional context, is a comparatively rare privilege. Scientists dabbling in science communication will need to demonstrate the *additional value* of the science that they wish to communicate beyond it being a mildly interesting “fun fact” or answer to a trivia question. This can be particularly challenging for fundamental research that does not have immediately apparent applications beyond its field.

One effective way of adding value to facts (“so what?”) is by infusing them with context and meaning through storytelling. For example, the background lore of most TV shows and movies are meaningless facts in reality, but valuable information to viewers invested in the wider story being told. Similarly, storytelling is a powerful tool for science communication: narratives increase comprehension, interest, and engagement in nonexpert audiences [25,26]. Even without a formal narrative structure, there is likely still some benefit from the inclusion of storytelling elements such as setting (e.g., vividly describe a key field site), conflict, or interesting characters (e.g., the scientist conducting the work, the model organism, or a person who may benefit from the study’s result).

## Rule 7: Tell your audience how to react

It may be appropriate to encourage a specific intellectual, behavioural, or emotional response (“call to action”) from the audience. For example, following a presentation at a city council meeting about high rates of road mortality in local turtles, the audience may be asked to avoid using a road near critical habitat during the breeding season, to volunteer, or to donate to a local wildlife rescue. Note that the call to action should be appropriate for the target audience as well as the tone and scope of the activity.

## Rule 8: Get some feedback, evaluate, and improve

Science communication is an iterative process involving feedback, evaluation, and modification [27]. Whenever possible, a science communicator should take the time to evaluate the success of the activity in achieving its original goal(s). A formal evaluation may not always be possible—some activities have very strict deadlines that preclude extensive preparation including goal development (e.g., comments or interviews in the media, unplanned interactions)—but informal personal reflection may still be valuable. Activities conducted in partnership with an institution may already have evaluation procedures in place, particularly if the organization is experienced in science communication and outreach.

Indicators of the success of an activity can include feedback such as self-assessment by the communicator or debriefing with the partner organization, quantitative metrics (attendance, views, reads, comments), a survey of audience members, or something else (see [28] for suggestions on how to evaluate workshops, much of which can be applied to science communication activities). Direct feedback from the audience of the activity is particularly valuable as it centres the audience experience, and in doing so may reveal issues not recognized by the communicator and improve the communicator’s understanding of their target audience. With the collected feedback, the communicator can now evaluate how well their activity succeeded in achieving its ultimate goal(s) and make modifications for future versions of the activity by revisiting each component of the activity.

## Rule 9: Consider equity, diversity, and inclusion

At its core, science communication can be understood as an exercise in inclusion, as it seeks to break down barriers to the access of scientific knowledge. Most science communication activities implicitly place the communicator, the scientist, as an authority figure due to their knowledge of the topic. It is key for the communicator to recognize this as well as other privileges that may be afforded to them (education, personal characteristics, etc.) and to use those privileges proactively in the service of the audience. For the communicator, this includes explicitly acknowledging the nature and limits of their expertise (e.g., avoid presenting unsettled science as settled), clearly distinguishing between scientific facts and personal opinions [29], and being aware of how their positionality impacts their perspective on science and science communication [30,31].

The communicator must also understand that just as science is political and does not occur in a vacuum, so is the communication of that science. Science communication and learning occurs in the intellectual, emotional, physical, social, and cultural context of each participant, and these contexts must be acknowledged, respected, and included in any science communication activity. For example, some communities are skeptical of scientific institutions due to negative historical and contemporary experiences with these institutions, and this context should inform how the communicator interacts with these communities [31]. Finally, the onus is on the communicator to ensure their activity is accessible to all by removing or minimizing as many potential barriers to full participation as possible.

## Rule 10: Remember that these rules are interdependent

**Fig 1:**
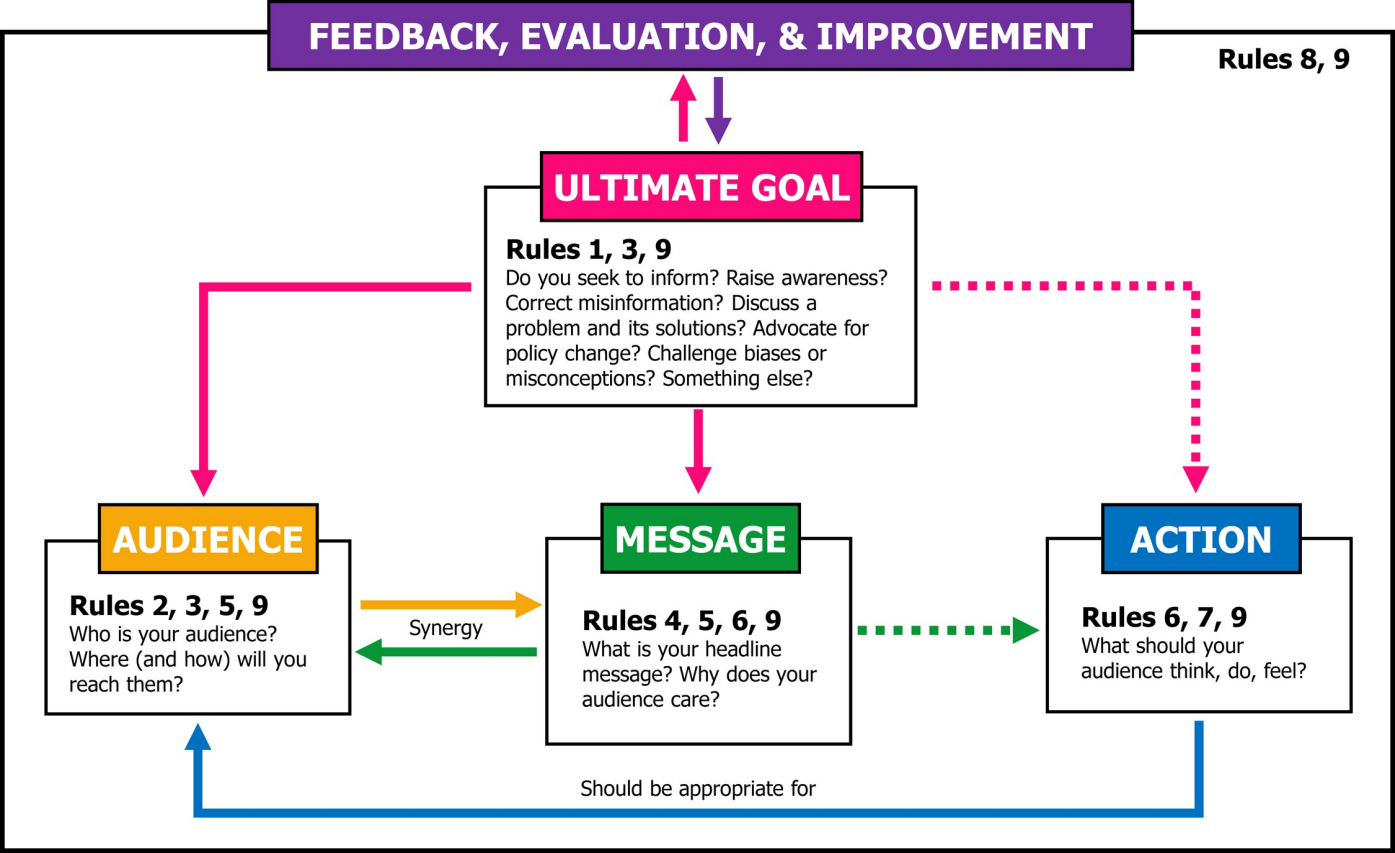
Integrated framework (Rule 10) for planning, developing, and evaluating science communication activities. The ultimate goal(s) of the activity inform its target audience, headline message, and, when appropriate, call to action. The goals of the activity are key to its evaluation and improvement in future iterations. Note that this framework may not capture all possible interactions between individual components.

A numbered list implies a hierarchy. But science communication activities are more than the sum of their individual parts, and integration of all of their underlying components is key for creating a coherent and effective contribution (e.g., in a framework) (Fig 1). For example, the ultimate goal(s) (Rule 1) inform both the design and completion of the activity (Rules 2 to 7, 9), as well as its evaluation (Rule 8). Decisions made at later steps (e.g., Rule 7) will feedback and modulate decisions made at earlier steps (Rule 2). Audience and message must also synergize with each other and are best developed somewhat simultaneously and not in strict sequence.
